# *Bifidobacterium* exopolysaccharides: new insights into engineering strategies, physicochemical functions, and immunomodulatory effects on host health

**DOI:** 10.3389/fmicb.2024.1396308

**Published:** 2024-05-06

**Authors:** Mahsa Sadeghi, Babak Haghshenas, Yousef Nami

**Affiliations:** ^1^Department of Food Biotechnology, Branch for Northwest and West Region, Agricultural Biotechnology Research Institute of Iran, Agricultural Research, Education and Extension Organization (AREEO), Tabriz, Iran; ^2^Regenerative Medicine Research Center (RMRC), Health Technology Institute, Kermanshah University of Medical Sciences, Kermanshah, Iran

**Keywords:** *Bifidobacterium*, exopolysaccharide, lactic acid bacteria, probiotics, immune system modulation

## Abstract

Bifidobacteria are a prominent type of bacteria that have garnered significant research attention for their exceptional probiotic properties and capacity to produce exopolysaccharides (EPSs). These compounds exhibit diverse physical, chemical, and biological characteristics, prompting numerous investigations into their potential applications. Researchers have noted their beneficial effects as immune modulators within the host’s body across various industries. Extensive research has been conducted on the immunomodulatory effects of bifidobacteria-derived EPSs, with emerging engineering strategies aimed at enhancing their immune-modulating capabilities. Understanding the structure, physicochemical properties, and biological activities of these compounds is crucial for their effective utilization across different industries. Our review encompassed numerous studies exploring *Bifidobacterium* and its metabolites, including EPSs, across various sectors, drawing from diverse databases. The distinctive properties of EPSs have spurred investigations into their applications, revealing their potential to bolster the immune system, combat inflammation, and treat various ailments. Additionally, these compounds possess antioxidant and antimicrobial properties, making them suitable for incorporation into a range of products spanning food, health, and medicine.

## 1 Introduction

Bifidobacteria, a genus within the Actinobacteria branch, are Gram-positive, anaerobic bacteria shaped like bacilli, acknowledged as a pivotal bacterial group in the intestinal microbial community, particularly during natural childbirth and infancy. First isolated in 1899 by Tissier from the feces of breastfed babies, this genus encompasses over 50 different species, including notable examples such as *Bifidobacterium bifidum*, *Bifidobacterium longum*, *Bifidobacterium breve*, *Bifidobacterium animal*, *Bifidobacterium adolescentis*, *Bifidobacterium pseudostreptococcus*, and *Bifidobacterium pseudolongum* (Schell et al., 2002; Fanning et al., 2012b; Eshaghi et al., 2017; Ruiz et al., 2017; Hidalgo-Cantabrana et al., 2018; Turroni et al., 2019; Yao et al., 2021; Li et al., 2023; Nicolescu et al., 2023; Pacyga-Prus et al., 2023).

In infants, bifidobacteria typically account for about 90% of intestinal bacteria, while in adults, this proportion decreases to 3%–5%. The mode of infant feeding significantly impacts the establishment of bifidobacteria in the gut, with breastfed infants showing higher levels compared to formula-fed counterparts (Liu et al., 2014; Ruiz et al., 2017; Choi et al., 2022; Li et al., 2023). These bacteria have the capability to produce metabolites known as exopolysaccharides (EPSs) during fermentation. Traditionally, EPSs play a vital role in fermented dairy products owing to its gelling and thickening properties, which also offer potential health benefits (Salazar et al., 2009; Sørensen et al., 2022). Due to their unique physiological and biological properties, these compounds can help strengthen the body’s immune system and effectively combat inflammation and various diseases, as well as serving as biological additives in various products such as food, pharmaceuticals, and health products (Xu et al., 2019; Angelin and Kavitha, 2020; Korcz and Varga, 2021; Netrusov et al., 2023).

In particular, EPSs, with their diverse chemical and structural composition, perform various functions across industries including agriculture, dairy, biofilm formation, cosmetics, etc., demonstrating their biotechnological significance (Netrusov et al., 2023). EPSs are a type of polysaccharide (Lim et al., 2020) and carbohydrate polymer widely distributed in various organisms, including plants, animals, microorganisms, and others (Yue et al., 2023a). EPS produced by lactic acid bacteria typically consists of glucose, galactose, and rhamnose sugar units in varying ratios, commonly found in genera such as bifidobacteria and lactobacilli (Kaur and Dey, 2023). These polysaccharides can either form a capsule when covalently attached to the cell surface, known as capsular polysaccharides (CPSs; Chen et al., 2017; Angelin and Kavitha, 2020; Alessandri et al., 2021), or be secreted into the surrounding environment of the cell, easily released in the growth environment, creating a slimy coating, and are then referred to as EPSs, which are crucial for the formation of bacterial biofilms (Alessandri et al., 2021; Xie et al., 2023).

The primary function of *Bifidobacterium* EPS is to shield these bacteria from acidity and bile salts during transit through the digestive system, thereby enhancing their adhesion to the intestinal mucosa (Llamas-Arriba et al., 2019). Consequently, Bifidobacteria are commonly utilized for the direct production of EPS in fermented products (Xu et al., 2019). Additionally, EPSs play various roles in protecting bacteria against osmotic stress (Bhagat et al., 2021), desiccation (Carezzano et al., 2023), extreme temperatures, salinity, UV rays, chemical agents such as antibiotics and heavy metals (Shukla et al., 2017), phagocytosis, and bacteriophage attacks (Laiño et al., 2016; Schmid, 2018; Angelin and Kavitha, 2020; Korcz and Varga, 2021; Netrusov et al., 2023). Hence, they serve as vital biological components in the interaction between microorganisms and the host (Laiño et al., 2016).

Certain species of *Bifidobacterium*, such as *B. longum*, *B. breve*, *B. bifidum*, *B. adolescentis*, *B. catenulatum*, and *B. infantis*, possess the capability to produce EPSs (Salazar et al., 2009; Hidalgo-Cantabrana et al., 2014b). These EPSs varieties exhibit a wide array of properties, serving as preservatives in food products, enhancing the immune system, acting as antimicrobial agents, and functioning as antioxidants (Fanning et al., 2012b; Laiño et al., 2016; Yue et al., 2023a). Moreover, they demonstrate antitumor potential (Wang et al., 2019), along with properties such as anti-diabetic, anti-inflammatory bowel, anti-aging, immune modulation, wound healing, and blood cholesterol reduction (Laiño et al., 2016; Llamas-Arriba et al., 2019; Yue et al., 2023a).

For instance, EPS derived from *B. longum* w11 has exhibited antioxidant activity *in vitro* and has been shown to regulate cellular oxidative stress (Inturri et al., 2017a). Furthermore, research indicates that bifidobacteria can mitigate the progression or symptoms of various diseases, including colorectal cancer, diarrhea, necrotizing enterocolitis, and inflammatory bowel disease (Liu et al., 2019). In a particular study, EPS isolated from lactobacilli and bifidobacteria demonstrated efficacy in attenuating the inflammatory response of enterotoxigenic *E. coli* on pig intestinal enterocytes (Wachi et al., 2014). Additionally, bifidobacteria have been observed to interact with human immune cells and modulate specific pathways involved in both innate and adaptive immune responses (Ruiz et al., 2017). By employing strategies such as optimizing cultivation conditions, as well as genetic and metabolic engineering, it becomes feasible to tailor the performance, structural, and functional characteristics of bacterial EPSs (Hidalgo-Cantabrana et al., 2014b).

## 2 Composition of *Bifidobacterium* EPS

EPS produced by bifidobacteria are recognized as potentially biologically active compounds. They exhibit a diverse range of structures and are primarily synthesized in response to various environmental stimuli (Nicolescu et al., 2023). Research indicates that the production rate of EPS by these bacteria is influenced by factors such as strain variation, environmental composition, and culture conditions, including temperature, pH, and carbon-nitrogen ratio (Angelin and Kavitha, 2020; Mohd Nadzir et al., 2021).

EPS can be categorized into two groups based on their properties, namely homopolysaccharides (HoPSs), composed of a single monosaccharide, and heteropolysaccharides (HePSs), consisting of one or more types of monosaccharides (Salazar et al., 2016; Angelin and Kavitha, 2020; Netrusov et al., 2023; Salimi and Farrokh, 2023). While homopolysaccharides are produced by certain lactic acid bacteria, their production in bifidobacteria remains unidentified (Hidalgo-Cantabrana et al., 2012; Castro-Bravo et al., 2018b; Lynch et al., 2018). In bifidobacteria, HePs consist of various repeating monosaccharide units, predominantly D-glucose, D-galactose, L-rhamnose, and occasionally, N-acetylated monosaccharides such as N-acetyl-glucosamine (GluNAc) and N-acetyl-galactosamine (GalNAc), as well as fucose, glucuronic acid, glycerol, or mannose, which may be branched or unbranched (Castro-Bravo et al., 2018b; Angelin and Kavitha, 2020; Jurášková et al., 2022; Netrusov et al., 2023).

Examples of heteropolysaccharides produced by bacteria include xanthan, alginate, valan, kefir, golan, and hyaluronic acid (Mohd Nadzir et al., 2021). The structure of the repeating units is elucidated using nuclear magnetic resonance and other chromatographic methods (Hidalgo-Cantabrana et al., 2012; Castro-Bravo et al., 2018b).

In a study investigating approximately 30 EPS from *Bifidobacterium* strains using various chromatographic methods, the main monosaccharides identified were D-galactose, found in all *Bifidobacterium* EPS, followed by D-glucose, present in over half of them, and finally L-rhamnose, found in half of the *Bifidobacterium* EPS. However, exceptions exist where the proportion of rhamnose is higher in certain *B. animalis* subsp. EPS (Hidalgo-Cantabrana et al., 2012). Additionally, studies indicate that *Bifidobacterium* strains containing D-mannose exhibit a higher rate of EPS production compared to L-mannose strains (Chen et al., 2017). Table 1 illustrating the structure of EPS units produced by several bifidobacteria strains.

**Table 1:** Unit structures of EPS synthesized by *Bifidobacterium* determined by NMR techniques.

Strain	NMR-structure^*^	Repeating unit size	Refs.
*B. adolescentis* CCDM 368	[→2)-β-D-Glcp-(1 → 3)-β-L-Rhap-(1 → 4)-β-D-Glcp-(1 → 3)α-L-Rhap-(1 → 4)-β-D-Glcp-(1 → 3)-α-D-Galp-(1→]	Hexasaccharide	Pacyga-Prus et al. (2023)
*B. animals* subsp. *lactic* IPLA-R1	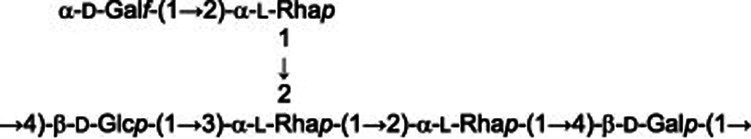	Hexasaccharide	Leivers et al. (2011)
*B. animals* subsp. *lactic* RH	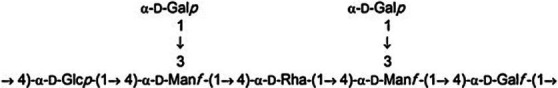	Heptasaccharide	Liu et al. (2014)
*B. infantis* ATCC 15697	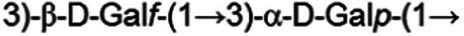	Disaccharide	Tone-Shimokawa et al. (1996)
*B. breve* YlT4010	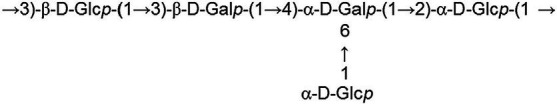	Pentasaccharide	Habu et al. (1987)
*B. longum* subsp. longum 35624™	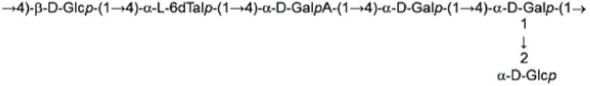	Hexasaccharide	Shang et al. (2013)
*B. longum* JBL05	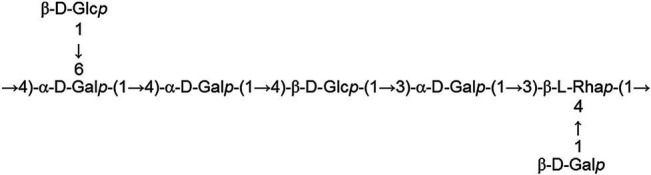	Heptasaccharide	Kohno et al. (2009)
*B. longum* W11	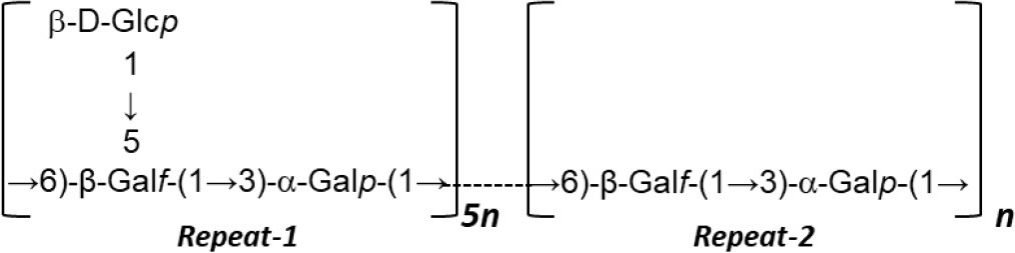	-	Inturri et al. (2017b)
*B. longum* YIT 4028	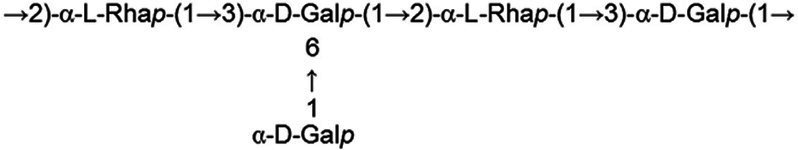	Pentasaccharide	Nagaoka et al. (1995)

^*^Gal, galactose; Glc, glucose; Mn, mannose; Rha, rhamnose; dTal, Deoxytalose; f, furanose ring conformation; p, pyranose ring conformation.

Both quantitative and qualitative methods are utilized to detect and identify EPS produced by bifidobacteria. Qualitative techniques encompass electron microscopy (EM) and confocal laser scanning microscopy (CLSM), providing visual insights into EPS structure and morphology. On the quantitative front, various methods are employed to analyze EPS composition including liquid chromatography (HPLC), gas chromatography (Han et al., 2016), colorimetric methods, size exclusion chromatography (SEC), ion exclusion chromatography (Konieczna et al., 2012), nuclear magnetic resonance spectroscopy (NMR), and Fourier transforms infrared spectroscopy (FTIR). These methods, either individually or in combination, enable comprehensive analyses of EPS composition, thereby contributing to a deeper understanding of their properties and potential applications (Korcz and Varga, 2021).

## 3 Pathway of EPS biosynthesis in *bifidobacteria*

The mechanism underlying EPS synthesis in the *Bifidobacterium* genus remains incompletely understood due to the elusive structure and composition of *Bifidobacterium* EPS. Previous studies have revealed that most *bifidobacteria* lack genes associated with HoPSs synthesis, which encode enzymes like glycan sucrase and fructan sucrase. However, a pathway for HePSs synthesis in bifidobacteria has been postulated based on the predicted functions of *eps* genes (Hidalgo-Cantabrana et al., 2014b; Castro-Bravo et al., 2018b).

HePSs polymers possess a complex composition, and their synthesis involves multiple enzymes and proteins, rendering the process intricate (Castro-Bravo et al., 2018b; Whitfield et al., 2020). Enzymes involved in HePSs biosynthesis can be categorized into four groups:

1. Hexokinase: These enzymes activate glucose to glucose-6-phosphate.
2. Uridine-5′diphosphate (UDP)-glucose pyrophosphorylase: They catalyze the conversion of glucose-1-phosphate to UDP-glucose, a critical molecule in EPS synthesis.
3. Glycosyltransferases: These enzymes transfer sugar nucleotides to a glycosyl carrier lipid.
4. Wzx protein (flipase) and ABC transporters: These groups of enzymes are involved in the polymerization and transport of EPS units across the cytoplasmic membrane. Wzx protein acts as a flipase, ejecting EPS repeat units bound to a lipid carrier across the membrane, while ABC transporters transport single repeating units attached to the lipid carrier UDP-C55 (Willis and Whitfield, 2013).

The synthesis of HePSs in bifidobacteria involves several steps:

1. Synthesis of repeating sugar units within the cytoplasm.
2. Cytoplasmic assembly of the EPS unit.
3. Export of the repeating EPS units to the extracellular side.
4. Polymerization and determination of the length of the final skeleton chain, with all steps except polymerization occurring in the cytoplasm (Cuthbertson et al., 2009; Zannini et al., 2016; Yang et al., 2019; Angelin and Kavitha, 2020).

Initially, glucose is converted into glucose-6-phosphate by intracellular hexokinase enzymes. Subsequently, glucose-6-phosphate is converted into glucose-1-phosphate by the enzyme phosphoglucomutase. UDP-glucose, essential for EPS synthesis, is then formed from glucose-1-phosphate by uridine diphosphate glucose pyrophosphorylase.

In the subsequent step, glycosyltransferase priming enzymes link the first monosaccharide from Pischas or the activated sugar nucleotide to a membrane-bound isoprenoid lipid carrier [Undecaprenyl phosphate (C55)]. Successive glycosyltransferases catalyze the glycosidic bond between new nucleotide sugars and the initial monosaccharide, leading to the addition of more sugar fragments. The structure of each oligopolysaccharide varies depending on the number and characteristics of Glycosyltransferases (Castro-Bravo et al., 2018a,b; Wang et al., 2019; Angelin and Kavitha, 2020).

Carrier lipids, identified as isoprenoid alcohols, have their terminal alcohol groups connected to remaining monosaccharides via a pyrophosphate bridge. These carrier lipids may undergo modifications such as acetylation, acylation, sulfosylation, and methylation, if necessary.

**Figure 1:**
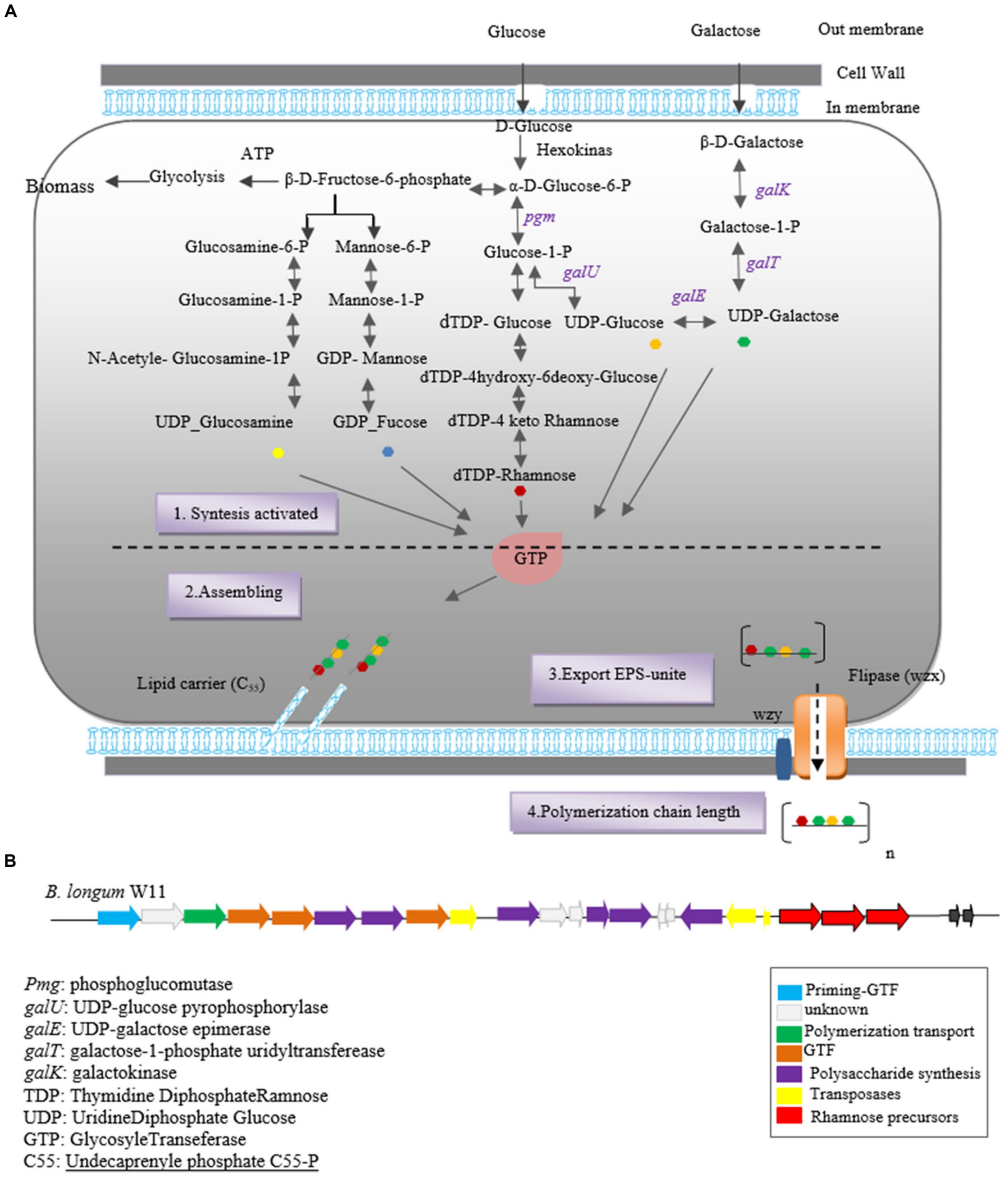
Heteropolysaccharide biosynthesis pathway in *Bifidobacterium* genus **(A)** and gene cluster of *Bifidobacterium longum* W11 **(B).**

Finally, the synthesized polymers are secreted to the extracellular side using two secretory systems: ABC transporters and the flippase-polymerase complex (WZX-WZY). Most eps clusters in *Bifidobacterium* strains indicate the existence of both systems in this genus. In the Wzx-Wzy-dependent pathway, the protein flippase (Wzx) ejects the EPS repeat units bound to the lipid carrier across the membrane, followed by a polymerase (Wzy) that transfers the repeating units outside the cell. The final chain length is determined by protein tyrosine kinase (Wzz; Hidalgo-Cantabrana et al., 2014b; Castro-Bravo et al., 2018a). A schematic representation of the hypothetical EPS biosynthesis pathway in Bifidobacterium, dependent on the Wzx-Wzy pathway, is depicted in Figure 1.

The biosynthesis of EPSs in *Bifidobacterium* species involves two key stages: the synthesis of precursor sugar nucleotides and the EPS cluster, which includes genes responsible for sugar nucleotide production and EPS biosynthesis enzymes. Notable genes involved in precursor sugar nucleotide synthesis include *galK, galE, galT, galU, rmlA, rmlB1*, and *rmlCD*, along with early glycosyltransferases. These genes have been identified in *B. longum* subspecies and strains NCC2705, DJO10A, and *B. longum* subsp. *longum* CRC 002 (Audy et al., 2010).

The *eps* gene cluster comprises genes encoding EPS biosynthesis enzymes and proteins. *In silico* analysis of the eps cluster has revealed a conserved gene set, including the predicted glycosyltransferase Primary (*p_gtf*), which catalyzes the synthesis of the EPS unit in the initial step. This gene is typically present in all eps clusters and is crucial for EPS production in the Bifidobacterium genus (Ferrario et al., 2016). For instance, the eps cluster of *B. longum* W11 has been described (Inturri et al., 2017b) as predicted in Figure 1B.

The first identification of EPS genes within the Bifidobacterium genome was reported by Lee and O’Mallivan (2010), uncovering predicted genes involved in EPS synthesis within these clusters (Castro-Bravo et al., 2018a). Subsequently, the sequence of the eps cluster responsible for EPS synthesis in *B. animalis* subsp. *lactis* was elucidated, along with the structure of the high molecular weight (HMW) rhamnose-rich repeating unit EPS produced by strain IPLA-R1 (Leivers et al., 2011).

Subsequent research and genetic analysis by Hidalgo et al. in 2014, focusing on 28 completed *Bifidobacterium* genomes, shed light on the diversity of the eps cluster among *Bifidobacterium* strains. For instance, *B. animalis* subsp. IPLA R1 was found to harbor a 54.3 kb eps cluster containing 42 genes, exhibiting variation across *Bifidobacterium* subspecies (Hidalgo-Cantabrana et al., 2014b; Ferrario et al., 2016). On the other hand, *B. bifidum* E3 strain’s eps cluster comprised 20 genes, indicating variation even within species (Yue et al., 2023a).

Moreover, higher diversity was observed in *B. adolescentis* and *B. breve* strains, highlighting strain-dependent variation in eps clusters. Notably, *B. breve* UCC2003 strain was found to possess three eps clusters, namely *eps1, eps2a*, and *eps2b* (Fanning et al., 2012a; Ferrario et al., 2016). However, despite the presence of a conserved eps2 cluster in the genome of *B. pullorum* LMG21816, this strain exhibited a negative EPS phenotype under tested conditions. It was suggested that the absence of *p-gtf* indicates the incompleteness of the eps cluster in this strain, resulting in the lack of EPS production (Ferrario et al., 2016).

In a study conducted by Ferrario et al. (2016), the genomes of 48 bifidobacteria strains available in the gene bank were analyzed to identify potential eps clusters. The researchers utilized the *p-gtf* sequence as a molecular marker to retrieve eps genome sequences, except for *B. bifidum* LMG11041. This study corroborated findings from a previous study by Hidalgo et al. in 2014, which indicated a lack of common structural organization in the eps biosynthesis clusters of bifidobacteria. However, the study revealed consistent interspecies diversity among strains possessing eps clusters, particularly in terms of cluster length, number, and predicted gene functions. The size of eps gene clusters varied significantly among Bifidobacterium strains, ranging from 9 genes identified in the eps region of *B. mongoliense* to 55 genes in *B. dentium* (Ferrario et al., 2016).

Notably, the glycosyltransferase enzyme, catalyzing the initial step of EPS synthesis, was found encoded within all *eps* clusters of the studied Bifidobacterium strains (Hidalgo-Cantabrana et al., 2014b; Castro-Bravo et al., 2018b; Zhou et al., 2019). However, interspecies variation in the genetic content responsible for EPS synthesis in *Bifidobacterium* indicates the flexibility of the genome within this genus (Zhou et al., 2019).

It’s noteworthy that there’s no common structural organization observed among species and strains within the *Bifidobacterium* genus. Furthermore, the G + C content of most EPS clusters in this genus are lower than that of the entire genome, suggesting horizontal acquisition of these genes. The likely donors of these genes are inhabitants with which bifidobacteria share a common environment, such as members of Lactobacillaceae and Lachnospiraceae. This suggests a dynamic evolutionary process shaping EPS synthesis within the *Bifidobacterium* genus (Chaib De Mares et al., 2015).

## 4 *Bifidobacterium* EPS engineering strategy

EPS engineering strategies aim to optimize the performance and unique properties of EPS for diverse applications in food, medicine, and industry, driven by insights into structure–function relationships. These strategies encompass interventions at the polymer synthesis stage through various treatments or at the biosynthesis level, thereby influencing structural composition. A straightforward approach to engineer EPS involves enzymatic modification, utilizing polysaccharide hydrolases and lyases, which serve as endoglycosidases or exoglycosidases. Typically sourced from microorganisms or their bacteriophages, these enzymes facilitate the alteration of EPS structure, enabling tailored functionalities (Boels et al., 2001).

Moreover, targeted manipulation of regulatory proteins offers another avenue to enhance EPS productivity. By augmenting the transcription of operons responsible for encoding EPS biosynthesis proteins, productivity can be significantly increased (Schmid et al., 2015). Additionally, EPS structures can be customized through modifications such as acetylation, phosphorylation, and sulfonation, enabling the attainment of desired functionalities. While these modifications have been extensively studied in lactobacillus strains, their exploration within the Bifidobacterium genus remains limited.

However, alternative strategies for modifying EPS in *Bifidobacterium* include altering molecular weight, adding or removing substituents and monomer sugars from side chains, and overexpressing genes encoding enzymes in EPS biosynthesis pathways. Additionally, housekeeping genes involved in sugar nucleotide formation play a crucial role. Given the abundance of the CRISPR-Cas system in bifidobacteria, gene editing using the CRISPR system offers a promising avenue for modifying EPS with new biological activities (Xu et al., 2019; Zuo et al., 2020; Sun and Zhang, 2021; Salimi and Farrokh, 2023).

In a study by Castro-Bravo et al. (2017), a novel double crossover recombination strategy was utilized in bifidobacteria. Specifically, they targeted the *Balate-1410* gene, which encodes a protein responsible for polymer chain elongation in *Bifidobacterium animalis* subsp. *lactis* DSM10140. Through this approach, they replaced the wild-type gene with a mutant variant, resulting in a mucoid phenotype. In essence, this research demonstrated that the ability to produce EPS in *B. animalis* DSM10140T could be reinstated by introducing a point mutation into the Balat_1410 gene, which plays a crucial role in EPS chain elongation. Analysis using NMR and SEC-MALS revealed that the mutant strain produced EPS with a higher molecular weight compared to the wild type. Furthermore, chemical and physical analyses confirmed the successful introduction of the mutation related to high molecular weight EPS in the recombinant strain (Castro-Bravo et al., 2017). This study showcases the potential of genetic manipulation techniques in enhancing EPS properties in *Bifidobacterium*, paving the way for future advancements in EPS engineering within this genus.

The impact of genes involved in nucleotide sugar production and priming glycosyltransferase (PGTF), which initiates the assembly of EPS repeat units by adding the first sugar-1-phosphate to a lipophilic carrier, is crucial in EPS engineering strategies. In a study conducted by Audy et al. (2010), the focus was on the expression of genes associated with sugar nucleotide production and EPS biosynthesis in *Bifidobacterium longum* subspCRC002. Their findings revealed that genes responsible for EPS biosynthesis were clustered within one or two transcription units, notably including PGTF, along with sugar nucleotide precursors for glucose, galactose, and rhamnose. Targeting these specific genes significantly influenced EPS production in this strain. Also *B. longum* subsp. CRC002 showed strong metabolic activity leading to increased production of EPS composed of glucose and galactose when PGTF-related genes were targeted. In addition, the expression of nucleotide sugar genes peaked at the exponential growth stage, indicating their importance in EPS biosynthesis. This study underscores the significance of targeting PGTF as a key enzyme in the biosynthetic pathway of EPS. By manipulating the expression of genes associated with nucleotide sugar production and PGTF, researchers can effectively enhance EPS production and tailor EPS composition to meet specific requirements for various applications (Audy et al., 2010).

In a study conducted by Hickey et al. (2021), they reported that strains of *Bifidobacterium breve* UCC2003 and *B. breve* JCM7017, which naturally produce EPS (WT), were compared with isogenic strains lacking EPS (EPS-mutants). The researchers observed that both WT strains lost their ability to produce EPS upon mutation, serving as positive controls for EPS deposition assays. Furthermore, the study investigated the impact of different carbohydrate sources on EPS production. Screening both *B. breve* strains in media containing glucose, lactose, and maltose revealed that while the WT strains did not precipitate EPS over a 6-h period, their EPS-mutants did. Additionally, the study explored the modulation of EPS on the cytokine response of Bone Marrow-Derived Macrophages (BMDM) and Dendritic Cells (BMDCs). When both EPS-isogenic strains were cultured with primary BMDM for 24 h, the absence of EPS from *B. breve* UCC2003 led to increased cytokine responses, with BMDM secreting TNF-α and IL10. Conversely, the absence of EPS from *B. breve* JCM7017 resulted in reduced cytokine responses. BMDCs did not exhibit significant TNF-α or IL10 production in response to any bacterial strain (Hickey et al., 2021).

## 5 Interaction between *Bifidobacterial* EPS and immune system

The immune system comprises two main components: innate and acquired immunity. The innate immune system, which is inherited and non-specific, serves to protect the host against microbial invasion and tissue damage (Ashraf and Shah, 2014; Hato and Dagher, 2015; Yatim and Lakkis, 2015). It operates with a short-term memory and includes various components such as the skin, phagocytic cells like macrophages, dendritic cells (DCs), neutrophils, and protein molecules like the complement and coagulation systems (Yatim and Lakkis, 2015; Kellie and Al-Mansour, 2017). In contrast, the acquired immune response is specific to target antigens and involves receptors expressed on B and T lymphocytes. This response becomes prominent a few days after encountering the antigen. Communication between the innate and adaptive immune systems is primarily facilitated by antigen-presenting dendritic cells (Yatim and Lakkis, 2015). Although the mechanisms of action of innate and acquired immunity differ, their cooperation is essential for mounting a fully effective immune response. This collaboration ensures a comprehensive defense against pathogens and other harmful agents (Hato and Dagher, 2015; Kellie and Al-Mansour, 2017).

The gastrointestinal tract (GIT) is home to a diverse array of microorganisms, including bacteria belonging to the genus *Bifidobacterium*, which play a crucial role in promoting host health.

In recent decades, the immunomodulatory capabilities of *Bifidobacterium* bacteria in interaction with human immune cells have garnered significant scientific interest. The modulating ability of strains within this bacterial genus has been linked to their production of EPSs (Hidalgo-Cantabrana et al., 2012). *Bifidobacterium* EPS exhibit anti-inflammatory and antimicrobial properties, contributing to their ability to regulate the immune system. Inflammation, which is a normal tissue repair process in response to infections and tissue damage, can lead to various inflammatory reactions such as pain, swelling, and fever due to the production of nitric oxide (NO) and prostaglandin E2 (PGE2; Angelin and Kavitha, 2020; Choi et al., 2022). Prolonged inflammation can result in excessive or insufficient production of pro-inflammatory cytokines, including IL-6 and TNF-α, and suppression of anti-inflammatory cytokines like IL-10, leading to inflammatory diseases and cancer (Angelin and Kavitha, 2020). *Bifidobacterium* EPSs have been demonstrated to modulate the inflammatory response of immune cells. For instance, EPS extracted and purified from *B. longum* BCRC 14634 exhibit mild immunomodulatory activity on J77A.1 macrophages by increasing IL-10 secretion and decreasing TNF-α levels (Wu et al., 2010). Other activities of EPS in modulating the host’s immune system include enhancing the proliferation of T and B lymphocytes, increasing natural killer (NK) cell activity, boosting the phagocytic capacity of mononuclear cells, inducing cytokine production, and enhancing overall host immune defense against pathogens (Angelin and Kavitha, 2020).

Moreover, *Bifidobacterium* plays a crucial role in reducing the risk of infection and preventing gastrointestinal cancers and inflammatory diseases such as inflammatory bowel disease (IBD; Rajoka et al., 2018). For instance, *B. longum* subsp. *longum* 35,624 has demonstrated clinical efficacy in irritable bowel syndrome, and comparison with a mutant derivative lacking EPS production illustrated the preventive role of EPS (Schiavi et al., 2016). Bifidobacterium EPS also exert their modulatory effects through antigen-presenting cells (APCs) or dendritic cells. EPS induce dendritic cells to secrete cytokines, leading to the differentiation of naïve T cells into regulatory T cells, which suppress inhibitory T cells, thereby promoting immune balance (Ashraf and Shah, 2014).

Various *in vitro* and *in vivo* models have been utilized to study the immunomodulatory activity of EPSs produced by *Bifidobacterium* strains. These models include peripheral blood mononuclear cells (PBMCs), mouse spleen cells, macrophage-like cell lines, and Gut Associated Lymphatic Tissues (GALT). Additionally, enterocytes such as the CaCo-2 or HT29 cell models have been employed in certain studies to investigate the immunomodulatory potential of Bifidobacterium bacteria due to their direct exposure to the intestinal environment, which could play a pivotal role in the initiation of *bifidobacteria*-host interactions (Ruiz et al., 2017).

*In vivo* models using human, mouse, and rat PBMCs have also been employed, and several instances of *in vitro* and *in vivo* models demonstrating immune responses are detailed in Table 2. The immunomodulatory capacity of *Bifidobacterium* EPS suggests potential health benefits for humans. However, differences in EPS structure and immune regulation between strains of the same *Bifidobacterium* species can lead to variations in their immunomodulatory effects. There is great structural variation in EPS polymers produced by bifidobacteria, even between strains of the same species (Hidalgo-Cantabrana et al., 2014b; Ferrario et al., 2016). In addition to differences in glycosidic bonds and degree of branching, changes in monosaccharide components and their amounts have been observed for different bifidobacteria strains (Hidalgo-Cantabrana et al., 2014b; Inturri et al., 2017b). This diversity could, in principle, lead to a large number of distinct EPS structures and theoretically to different immunomodulatory effects on the host (Hickey et al., 2021). For instance, *B. breve* UCC2003, with a thicker EPS layer, exhibits a more anti-inflammatory phenotype compared to *B. breve* JCM7017, as evidenced by modulation of macrophage IL-10 and TNFα and dendritic cell expression of *Tnfa*, *Il6*, *Il12a*, and *Il23a*. Murine *B. pseudolongum* UMB287 MBP-01 EPS increases intestinal Tregs, whereas porcine-derived *B. pseudolongum* ATCC25526 EPS does not. However, EPS from both strains lead to increased dendritic cells (DCs), mesenteric lymph node (MLN) DCs, and intestinal MLN macrophages (Hickey et al., 2021; Gavzy et al., 2023).

**Table 2:** *In-vitro* and *in-vivo* models to study the *Bifidobacterium* EPSs immunomodulatory potential.

No.	Models	Bifidobacterium strain	Immune index	Refs.
***In-vitro***				
1	Human PBMC	*B. longum* W11	INF-α, IL1-β, IL-10, IL-6	Inturri et al. (2017a)
2	Naïve rats (GAIT)	*B. longum* subsp. *lactis*	TNF-α/ Il-10, TNF-α/TGFβ, IFN-γ/IL-17, IFN-γ/IL-4	Hidalgo-Cantabrana et al. (2014a)
3	Naïve rats (PBMC)	*B. longum* subsp. *lactis*	TNF-α/ Il-10, TNF-α/TGFβ, IFN-γ/IL-17, IFN-γ/IL-4	Hidalgo-Cantabrana et al. (2014a)
4	OVA-sensitized mice	*B. adolescentis* CCDM 368	IL-4, Il-5, Il-13, Il-10, TFN-γ	Pacyga-Prus et al. (2023)
5	Macrophages mice (J77A.1)	*B. longum* BCRC 14634	IL-10, TFNα	Wu et al. (2010)
6	Splenocytes macrophages	*B. longum* KACC 91563	TNF-*α*, IgE, IL-2, 4, 6, 10, IFN-*γ*	Choi et al. (2019)
7	RAW264.7 cell	*B. longum* Bif10 and Bif16	TNF-*α*, IL-1*β*, IL-6, SCFA	Singh et al. (2020)
8	Mice splenocytes	*B. breve* ucc2003	INF-α, TNFα, IL-12	Fanning et al. (2012a)
9	Human PBMC	*B. animalis* subsp. *lactis* A1		Hidalgo-Cantabrana et al. (2012) and Lopez et al. (2012)
		*B. animalis* subsp. *lactis* A1dOx		
		*B. animalis* subsp. *lactis* A1dOxR		
10	RAW264.7 macrophages or mice splenocytes	*B. adolescentis* IF1-03	IL-6, IL-10, TGFβ, Treg	Yu et al. (2019)
11	Human PBMC	*B. animalis* RH	IL-1α, IFN-γ, TNF-β, IL-17	Xu et al. (2017)
***In-vivo***				
1	Wister rats	*B. animal* subsp*. lactic* IPLA-R1	TGFβ, IL-6	Salazar et al. (2014)
2	Mice	*B. breve* ucc2003	INF-γ, IL-12, TNF-α	Fanning et al. (2012a)
3	Human	*B. longum* 536	INF-γ, IL-4	Wu et al. (2010)
4	Mice	*B. adolescentis* ATCC15703	TNF-α, IL-6, IL-1β, IL-18, IL-22, IL-9, IL-10, IL-4, Il-5, Treg	Fan et al. (2021)
5	Human	*B. infantis* 35,624	Il-10, Foxp3	Konieczna et al. (2012)
6	Mice	*B. bifidum* BGN4	MCP-1, TNF-α, IFN-γ, CD	Ku et al. (2016)
7	CY-induced immunosuppressed mice	*B. animalis* RH	IFN-γ, IL-2, IL-10 and IgG	Xu et al. (2017)
8	Mice	*B. longum* Bif10 and Bif16	SCFA, TNF-α, IL-1β, IL-6, IL-1	Singh et al. (2020)
9	Mice	*B. longum* ATCC 15707	TNF-α, IL-6, TGF-β	Celiberto et al. (2017)

The modulatory effects of *Bifidobacterium* EPS depend on factors such as molecular weight and chemical composition. Generally, EPS with low molecular weight induce higher levels of cytokines, while those with high molecular weight induce a lower cytokine secretion or decrease the TNFα/IL-10 ratio, indicating an anti-inflammatory effect (Lopez et al., 2012; Salazar et al., 2014). For example, EPS from *B. animalis* subsp. *lactis* IPLA-R1, with a high rhamnose percentage and molecular weight, increases IL-10 production in PBMC models and decreases TNFα production in human colon biopsies (Hidalgo-Cantabrana et al., 2015). Moreover, studies have shown correlations between EPS polymer composition, structure, and size, and the corresponding immune response, suggesting that the physical and chemical characteristics of EPS influence their immunomodulatory properties (Lopez et al., 2012).

Similar findings were reported in a study involving co-incubation of purified EPS from three strains of *B. animalis* (A1, A1dOx, and A1dOxR) with PBMCs. The analysis revealed that the A1dOxR extracellular polymer caused less release of pro- and anti-inflammatory cytokines compared to other strains, attributed to its higher molecular weight. Additionally, EPS from *Bifidobacterium adolescentis* strains IF1-11 and IF1-03, with high molecular weight, induced different cytokine profiles when co-cultured with macrophage RAW264.7 and mouse spleen cells, with strain IF1-03 exhibiting anti-inflammatory effects, while strain IF1-11 showed pro-inflammatory effects (Alessandri et al., 2019).

Dendritic cells (DCs) represent a specialized subset of myeloid cells that respond to infection by capturing antigens, processing them into smaller peptides, and subsequently presenting them to T lymphocytes. As primary antigen-presenting cells (APCs), DCs serve as a crucial link between innate and adaptive immunity. Both mouse dendritic cells and human blood mononuclear cells hold promise for future investigations into the modulatory potential of EPSs derived from *Bifidobacterium* strains.

*In vitro* studies involving the treatment of DCs with *Bifidobacterium bifidum* PRI1, followed by co-culture with naïve CD4 T cells, have demonstrated enhanced induction of regulatory T cells (Tregs) and production of interleukin-10 (IL-10; Gavzy et al., 2023). These findings underscore the immunomodulatory capabilities of *Bifidobacterium* EPS, particularly in promoting immune tolerance and anti-inflammatory responses mediated by Tregs and IL-10. Such studies pave the way for further exploration of the interaction between *Bifidobacterium*-derived EPS and DCs, shedding light on their potential therapeutic applications in immune-related disorders.

One of the extensively researched aspects of immune system modulation by probiotic bacteria is the regulation of cytokine production. Cytokines, which are protein molecules synthesized by immune cells, play diverse roles in defense mechanisms, including inflammation, B and T lymphocyte differentiation, immune system activation, and eradication of foreign antigens. Additionally, cytokines can significantly contribute to the pathogenesis of autoimmune and immune-mediated kidney diseases (Yatim and Lakkis, 2015). Probiotic bacteria can induce the secretion of cytokines from intestinal epithelial cells in a strain-specific manner. For instance, in a study involving mice treated with *Bifidobacterium adolescentis* ATCC15703, lower levels of pro-inflammatory cytokines such as TNFα, IL-6, IL-1β, IL-18, IL-22, and IL-9 were observed compared to the control group. Conversely, higher levels of the anti-inflammatory cytokine IL-10 and the cytokines IL-4 and IL-5, along with increased regulatory T cells (Tregs), were detected in the colons of colitis mice receiving *B. adolescentis* ATCC15703 (Gavzy et al., 2023). These findings underscore the strain-specific immunomodulatory effects of probiotic bacteria and highlight their potential therapeutic implications in inflammatory conditions.

The impact of EPS produced by *Bifidobacterium longum* W11 on the immune response of peripheral blood mononuclear cells (PBMCs), both with and without ConA stimulation, was investigated. Specifically, in unstimulated PBMCs, EPS induced the production of IL-6 at higher concentrations and IL-10 only at lower concentrations. Moreover, when PBMCs were stimulated with ConA, EPS increased the production of various cytokines, except for IL-10 (Inturri et al., 2017a). Cytokines serve as soluble mediators of host defense responses, playing crucial roles in both specific and non-specific mechanisms for eliminating foreign antigens (Ashraf and Shah, 2014). The cell surface components of *B. longum* strains NCC 2705, ATCC 15707, and BIF53 have been shown to stimulate the production of IL-10 and TNFα in isolated peripheral blood mononuclear cells (Gavzy et al., 2023).

Macrophages play a crucial role in recognizing bacteria and, upon activation by microbial metabolites like polysaccharides, they engage in bacterial killing through phagocytosis, secrete cytokines for immune modulation, and present antigens to helper T cells. Notably, intestinal macrophages exhibit restrained proinflammatory cytokine production in response to various inflammatory stimuli, including microbial components (Wu et al., 2010).

In a study investigating the immunomodulatory and anti-inflammatory properties of EPSs from *B. longum* subsp. *infantis* E4, conducted on RAW264.7 cells, spleen lymphocytes, and mouse NK cells *in vitro*, it was found that EPS enhanced the growth and phagocytic activity of RAW264.7 macrophages, increased spleen lymphocyte proliferation, and boosted NK cell activity. These findings suggest that EPS derived from *B. infantis* E4 possesses immune-modulating and anti-inflammatory properties, potentially serving as a prebiotic for promoting future health maintenance. Hence, EPS from *B. longum* subsp. *infantis* E4 could be considered a functional food ingredient with modulatory and anti-inflammatory effects on immune cells, thus broadening the scope of immune modulators (Yue et al., 2023b).

In another study, EPS produced by *B. longum* strain BCRC 14634 was observed to induce increased production of the anti-inflammatory cytokine IL-10 by murine macrophages, compared to baseline conditions. Additionally, the presence of EPS was associated with lower levels of the pro-inflammatory cytokine TNFα, compared to lipopolysaccharides (Wu et al., 2010).

## 6 Application of *Bifidobacterium* EPS in the food industry

Beneficial microorganisms like lactic acid bacteria and bifidobacteria possess the capability to produce postbiotic bioactive substances, including EPSs. Leveraging their technological advantages, they are extensively employed as starter cultures in the production of fermented food products (Xu et al., 2019), as well as functional foods (Sørensen et al., 2022), which can directly or indirectly impact human health (Castro-Bravo et al., 2018b; Xu et al., 2019).

In the food industry, these microorganisms serve as functional additives, contributing to the production of products with desirable attributes. Particularly in the dairy sector, EPSs function as thickeners, emulsifiers, and stabilizers without imparting unpleasant tastes. They prevent water separation in cheese, resulting in a softer and creamier product, thereby increasing cheese yield. Moreover, they enhance yogurt viscosity and water holding capacity (Xu et al., 2019).

Another notable application of EPS is in bakery products, where it increases bread volume and moisture content, resulting in a softer texture for both gluten-containing and gluten-free bread. Additionally, EPSs mitigate staling by impeding starch retrogradation, thereby improving shelf life owing to their water-binding properties (Xu et al., 2019). However, despite their numerous advantages, EPSs can also have detrimental effects. For instance, EPS-producing bacteria can spoil alcoholic beverages like beer and wine. Furthermore, EPS synthesis and the formation of intestinal plaque and biofilm can lead to health issues in the food industry.

As bifidobacteria are commonly utilized as probiotics in dairy products, there is a growing interest in exploring the potential of EPS-producing *Bifidobacterium* strains as functional starters for low-fat yogurt production (Prasanna et al., 2013). Both lactic acid bacteria and bifidobacteria are known to produce EPS, contributing to the texture and mouthfeel of yogurt, a popular fermented milk product.

In the context of health-conscious consumers preferring low-fat dairy options, the production of low-fat yogurt presents certain challenges, such as compromised texture and taste, characterized by high synthesis and low viscosity. While thickeners can address these issues, regulations prohibiting the addition of stabilizers in yogurt have led to the exploration of EPSs as viable alternatives in the European Union (Xu et al., 2019).

The texture of yogurt is influenced by various factors including milk heating, pH, fermentation duration, milk composition (particularly protein or fat content), as well as the concentration and structure of EPS produced by the starter culture, which plays a pivotal role in yogurt gelation. Thus, the selection of strains with higher EPS production is crucial. However, studies indicate a weak correlation between EPS quantity and resulting effects on rheological properties, underscoring the significance of EPS structures (e.g., monosaccharide composition, charge, molecular weight, degree of branching, and backbone stiffness) and interactions between EPSs and milk components, especially proteins, in determining yogurt texture (Gentès et al., 2011; Han et al., 2016). Key textural characteristics of yogurt, including viscosity, syneresis (whey separation), gelation pH, and gel firmness, are considered essential factors in product quality and consumer acceptance (Xu et al., 2019).

The EPS produced by *B. infantis* CCUG 52486 and *B. infantis* NCIMB 702205 exhibits notable emulsification activity and favorable rheological properties, leading to enhanced viscosity in fermented low-fat milk. Among these strains, *B. longum* subsp. *infantis* CCUG 52486 stands out as particularly promising. It can be effectively incorporated into yogurt starter cultures to produce low-fat yogurt with probiotic benefits while simultaneously improving the physicochemical and rheological characteristics of the product (Prasanna et al., 2013).

Presently, there are 39 species of lactic acid bacteria and 5 species of bifidobacteria that have been granted Generally Recognized as Safe (GRAS) status by the European Food Safety Authority (Hazards et al., 2022). These strains are also included in the Qualified Presumption of Safety (QPS) list, making their application in food matrices more accessible. However, it’s worth noting that neither the EFSA nor the FDA have established any health claims for the use of EPS from lactic acid bacteria in food products (Hazards et al., 2022).

## 7 Conclusion and future perspective

In recent years, there has been growing interest in the EPS produced by *Bifidobacterium* bacteria. EPS serve as a crucial external protection and covering for *Bifidobacterium*, offering resilience against the surrounding environment. The synthesis of EPS involves a complex interplay of molecules, proteins, and enzymes, including glycosyl transferase and polymerases. The structural and chemical characteristics of EPS determine their diverse functions, rendering them beneficial in various industries such as agriculture, dairy, cosmetics, and pharmaceuticals.

*In silico* analysis conducted on available *Bifidobacterium* genomes has revealed a lack of consensus structural organization in EPS clusters, unlike those identified in LAB-*eps* clusters. However, some common features of bifido-*eps* clusters, such as high inter- and intraspecific organizational diversity, are observed, with the exception of *B. animalis* subsp. *lactis*. Additionally, the EPS cluster generally exhibits a lower G + C content compared to the entire bifidobacterial genome. The increasing availability of genomes in the future will offer researchers opportunities for genetic and metabolic engineering to tailor EPS production for use in the food or pharmaceutical industries.

Despite the growing interest, limited research has been conducted on *in vitro* and *in vivo* models to assess the immunomodulatory activity of EPS-producing bifidobacteria. Further scientific investigation is needed to enhance EPS efficiency and conduct *in vivo* studies to explore their therapeutic properties fully. This will enable researchers to harness the full potential of EPS in various applications.

## Author contributions

MS: Writing – original draft. BH: Conceptualization, Project administration, Writing – original draft. YN: Writing – review & editing.
